# 3-Methyl-1-(prop-2-en-1-yl)quinoxalin-2(1*H*)-one

**DOI:** 10.1107/S1600536810023640

**Published:** 2010-06-23

**Authors:** Youssef Ramli, Rachid Slimani, Hafid Zouihri, Saïd Lazar, E. M. Essassi

**Affiliations:** a Laboratoire Nationale de Contrôle des Médicaments, Direction du Médicament et de la Pharmacie, BP 6206, 10000 Rabat, Morocco; b Laboratoire de Biochimie, Environnement et Agroalimentaire (URAC 36), Faculté des Sciences et Techniques Mohammedia, Université Hassan II Mohammedia-Casablana, BP 146, 20800 Mohammedia, Morocco; c Laboratoires de Diffraction des Rayons X, Division UATRS, Centre National pour la Recherche Scientifique et Technique, Rabat, Morocco; d Laboratoire de Chimie Organique Hétérocyclique, Université Mohammed, V-Agdal, BP 1014, Rabat, Morocco

## Abstract

In the mol­ecule of the title compound, C_12_H_12_N_2_O, the quinoxaline ring is planar with an r.m.s. deviation of 0.007 (15) Å. The dihedral angle between the quinoxaline and propenyl planes is 82.1 (2)°. The crystal packing is stabilized by offset π–π stacking between the quinoxaline rings [centroid–centroid distance = 3.8832 (9) Å].

## Related literature

For biological activity of quinoxaline derivatives, see: Kleim *et al.* (1995 ▶). For their anti­tumor, and anti­tuberculous properties, see: Abasolo *et al.* (1987 ▶); Rodrigo *et al.* (2002 ▶). For the anti­­fungal, herbicidal, anti­dyslipidemic and anti-oxidative activities of quinoxaline derivatives, see: Jampilek *et al.* (2005 ▶); Sashidhara *et al.* (2009 ▶); Watkins *et al.* (2009 ▶). For bond-length data, see: Allen *et al.* (1987 ▶).
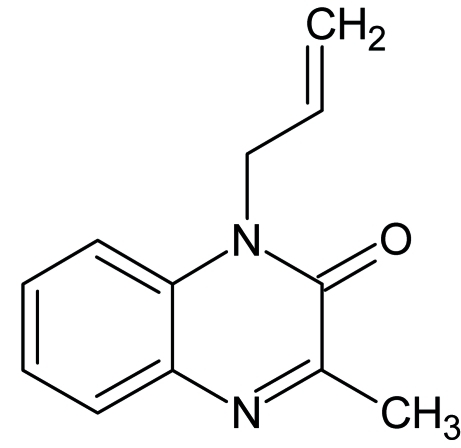


## Experimental

### 

#### Crystal data

C_12_H_12_N_2_O
*M* *_r_* = 200.24
Monoclinic, 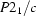

*a* = 5.0722 (5) Å
*b* = 13.4707 (13) Å
*c* = 15.0507 (13) Å
β = 95.082 (5)°
*V* = 1024.31 (17) Å^3^
*Z* = 4
Mo *K*α radiation
μ = 0.09 mm^−1^
*T* = 296 K
0.32 × 0.31 × 0.13 mm

#### Data collection

Bruker X8 APEXII CCD area-detector diffractometer
11850 measured reflections
2546 independent reflections
1726 reflections with *I* > 2σ(*I*)
*R*_int_ = 0.049

#### Refinement

*R*[*F* ^2^ > 2σ(*F* ^2^)] = 0.051
*wR*(*F* ^2^) = 0.151
*S* = 1.08
2546 reflections
137 parameters
H-atom parameters constrained
Δρ_max_ = 0.23 e Å^−3^
Δρ_min_ = −0.17 e Å^−3^

### 

Data collection: *APEX2* (Bruker, 2005 ▶); cell refinement: *SAINT* (Bruker, 2005 ▶); data reduction: *SAINT*; program(s) used to solve structure: *SHELXS97* (Sheldrick, 2008 ▶); program(s) used to refine structure: *SHELXL97* (Sheldrick, 2008 ▶); molecular graphics: *ORTEPIII* (Burnett & Johnson, 1996 ▶), *ORTEP-3 for Windows* (Farrugia, 1997 ▶) and *PLATON* (Spek, 2009 ▶); software used to prepare material for publication: *WinGX* (Farrugia, 1999 ▶) and *publCIF* (Westrip, 2010 ▶).

## Supplementary Material

Crystal structure: contains datablocks I, New_Global_Publ_Block. DOI: 10.1107/S1600536810023640/dn2579sup1.cif

Structure factors: contains datablocks I. DOI: 10.1107/S1600536810023640/dn2579Isup2.hkl

Additional supplementary materials:  crystallographic information; 3D view; checkCIF report

## Figures and Tables

**Fig. 1.**
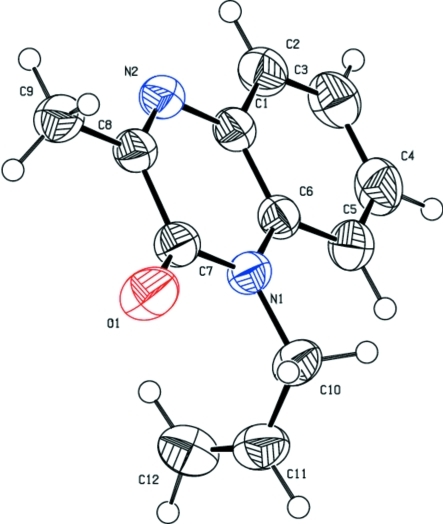
: Molecular structure of the title compound with the atom-labelling scheme. Displacement ellipsoids are drawn at the 50% probability level. H atoms are represented as small spheres of arbitrary radii.

**Fig. 2.**
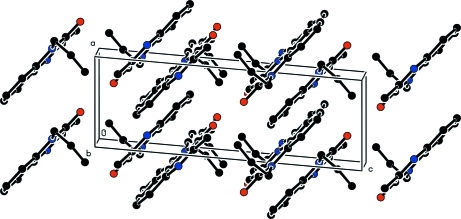
: Packing view of the crystal structure of the title compound.

## supplementary crystallographic information

### Comment

Quinoxaline derivatives are a very important class of nitrogen-containing compounds and have been widely used in dyes, pharmaceuticals and electrical/photochemical materials. Quinoxaline ring moiety constitute part of the chemical structures of various antibiotics such as Echinomycin, Levomycin and Actinoleutin that are known to inhibit growth of gram positive bacteria and are active against various transplantable tumors.

Quinoxaline derivatives were found to exhibit antimicrobial [Kleim *et al.* 1995], antitumor [Abasolo *et al.* 1987], and antituberculous activity [Rodrigo *et al.*2002]. They, also, exhibit interesting antifungal, herbicidal, Antidyslipidemic and antioxidative activities of quinoxaline derivatives, see: (Jampilek *et al.* 2005, Sashidhara *et al.* 2009, Watkins *et al.* 2009).

The dihedral angle between the quinoxaline and propenyl planes is 82.1 (2) (Fig. 1). Bond lengths and angles in title molecule are normal (Allen *et al.*, 1987). The crystal packing is stabilized by offset π-π stacking between the quinoxalin rings.

### Experimental

To a solution of 3-methylquinoxali-2(1*H*)-one (1 g) in 20 ml of dimethylformamide was added allylchloride (0.85 ml),K2CO3 (0.95 g) and catalytic amont of tetrabutylammonium bromide.The mixture was stirred at room temperature for 24 h.Then the solvent was remdove under reduce pressure,the residue was cristallized in ethanol to afford the product.

### Refinement

Although found in a difference map, H atoms were introduced in calculated positions and treated as riding with C—H = 0.96 Å for methyl groups, C—H = 0.93 Å for aromatic and C—H = 0.97 Å for methine with U iso (H) = 1.2U_eq_ (aromatic, methine ) or U iso (H) = 1.5U_eq_ (methyl).

### Crystal data

**Table:**

C_12_H_12_N_2_O	*F*(000) = 424
*M**_r_* = 200.24	*D*_x_ = 1.298 Mg m^−^^3^
Monoclinic, *P*2_1_/*c*	Melting point: 1486 K
Hall symbol: -P 2ybc	Mo *K*α radiation, λ = 0.71073 Å
*a* = 5.0722 (5) Å	Cell parameters from 2764 reflections
*b* = 13.4707 (13) Å	θ = 2.4–27.4°
*c* = 15.0507 (13) Å	µ = 0.09 mm^−^^1^
β = 95.082 (5)°	*T* = 296 K
*V* = 1024.31 (17) Å^3^	Block, colourless
*Z* = 4	0.32 × 0.31 × 0.13 mm

### Data collection

**Table:**

Bruker X8 APEXII CCD area-detector diffractometer	1726 reflections with *I* > 2σ(*I*)
Radiation source: fine-focus sealed tube	*R*_int_ = 0.049
graphite	θ_max_ = 28.3°, θ_min_ = 2.7°
φ and ω scans	*h* = −6→6
11850 measured reflections	*k* = 0→17
2546 independent reflections	*l* = 0→20

### Refinement

**Table:**

Refinement on *F*^2^	Primary atom site location: structure-invariant direct methods
Least-squares matrix: full	Secondary atom site location: difference Fourier map
*R*[*F*^2^ > 2σ(*F*^2^)] = 0.051	Hydrogen site location: inferred from neighbouring sites
*wR*(*F*^2^) = 0.151	H-atom parameters constrained
*S* = 1.08	*w* = 1/[σ^2^(*F*_o_^2^) + (0.0723*P*)^2^ + 0.0888*P*] where *P* = (*F*_o_^2^ + 2*F*_c_^2^)/3
2546 reflections	(Δ/σ)_max_ = 0.001
137 parameters	Δρ_max_ = 0.23 e Å^−^^3^
0 restraints	Δρ_min_ = −0.17 e Å^−^^3^

### Special details

**Table:**

Experimental. The data collection nominally covered a sphere of reciprocal space, by a combination of seven sets of exposures; each set had a different φ angle for the crystal and each exposure covered 0.5° in ω and 30 s in time. The crystal-to-detector distance was 37.5 mm.
Geometry. All esds (except the esd in the dihedral angle between two l.s. planes) are estimatedusing the full covariance matrix. The cell esds are taken into account individually in the estimation of esds in distances, angles and torsion angles; correlations between esds in cell parameters are only used when they are defined by crystal symmetry. An approximate (isotropic) treatment of cell esds is used for estimating esds involving l.s. planes.
Refinement. Refinement of *F*^2^ against ALL reflections. The weighted *R*-factor *wR* and goodness of fit *S* are based on *F*^2^, conventional *R*-factors *R* are based on *F*, with *F* set to zero for negative *F*^2^. The threshold expression of *F*^2^ > σ(*F*^2^) is used only for calculating *R*-factors(gt) *etc*. and is not relevant to the choice of reflections for refinement. *R*-factors based on *F*^2^ are statistically about twice as large as those based on *F*, and *R*- factors based on ALL datawill be even larger.

### Fractional atomic coordinates and isotropic or equivalent isotropic displacement parameters (Å^2^)

**Table:**

	*x*	*y*	*z*	*U*_iso_*/*U*_eq_	
O1	−0.3390 (2)	0.34712 (9)	0.06022 (9)	0.0657 (4)	
N1	−0.0193 (2)	0.29591 (9)	0.16502 (8)	0.0408 (3)	
N2	0.1311 (2)	0.49458 (9)	0.18738 (8)	0.0445 (3)	
C1	0.2649 (3)	0.41959 (11)	0.23590 (9)	0.0408 (3)	
C2	0.4769 (3)	0.44552 (13)	0.29645 (10)	0.0514 (4)	
H2	0.5253	0.5119	0.3030	0.062*	
C3	0.6150 (3)	0.37488 (15)	0.34638 (11)	0.0589 (5)	
H3	0.7560	0.3931	0.3868	0.071*	
C4	0.5434 (3)	0.27599 (15)	0.33625 (11)	0.0579 (5)	
H4	0.6364	0.2278	0.3704	0.069*	
C5	0.3375 (3)	0.24845 (13)	0.27651 (11)	0.0503 (4)	
H5	0.2932	0.1817	0.2698	0.060*	
C6	0.1937 (3)	0.31975 (11)	0.22568 (9)	0.0395 (3)	
C7	−0.1541 (3)	0.36731 (11)	0.11482 (10)	0.0441 (4)	
C8	−0.0643 (3)	0.47053 (11)	0.13088 (9)	0.0424 (4)	
C9	−0.2158 (3)	0.54800 (12)	0.07845 (11)	0.0543 (4)	
H9A	−0.3878	0.5548	0.0997	0.081*	
H9B	−0.2343	0.5293	0.0167	0.081*	
H9C	−0.1233	0.6101	0.0850	0.081*	
C10	−0.1160 (3)	0.19385 (11)	0.15522 (11)	0.0483 (4)	
H10A	−0.3032	0.1956	0.1355	0.058*	
H10B	−0.0975	0.1621	0.2133	0.058*	
C11	0.0207 (3)	0.13211 (13)	0.09201 (12)	0.0578 (5)	
H11	−0.0243	0.0652	0.0896	0.069*	
C12	0.1942 (4)	0.16040 (15)	0.04015 (13)	0.0669 (5)	
H12A	0.2467	0.2265	0.0398	0.080*	
H12B	0.2672	0.1147	0.0030	0.080*	

### Atomic displacement parameters (Å^2^)

**Table:**

	*U*^11^	*U*^22^	*U*^33^	*U*^12^	*U*^13^	*U*^23^
O1	0.0682 (8)	0.0524 (8)	0.0700 (8)	−0.0060 (6)	−0.0296 (7)	0.0027 (6)
N1	0.0443 (6)	0.0358 (7)	0.0413 (6)	−0.0016 (5)	−0.0024 (5)	0.0006 (5)
N2	0.0518 (7)	0.0402 (7)	0.0407 (6)	−0.0005 (5)	−0.0013 (5)	−0.0018 (5)
C1	0.0435 (7)	0.0433 (9)	0.0353 (7)	0.0004 (6)	0.0020 (6)	−0.0008 (6)
C2	0.0527 (9)	0.0550 (10)	0.0452 (8)	−0.0061 (7)	−0.0038 (7)	−0.0033 (7)
C3	0.0527 (9)	0.0755 (13)	0.0458 (9)	−0.0016 (8)	−0.0107 (7)	−0.0006 (8)
C4	0.0592 (10)	0.0648 (12)	0.0474 (9)	0.0098 (8)	−0.0080 (7)	0.0103 (8)
C5	0.0568 (9)	0.0471 (9)	0.0460 (8)	0.0046 (7)	−0.0014 (7)	0.0053 (7)
C6	0.0416 (7)	0.0415 (9)	0.0352 (7)	0.0006 (6)	0.0026 (6)	−0.0005 (6)
C7	0.0464 (8)	0.0423 (9)	0.0420 (8)	0.0003 (6)	−0.0045 (6)	−0.0002 (6)
C8	0.0487 (8)	0.0398 (8)	0.0380 (7)	0.0027 (6)	0.0003 (6)	−0.0005 (6)
C9	0.0653 (10)	0.0439 (9)	0.0518 (9)	0.0069 (7)	−0.0059 (8)	0.0015 (7)
C10	0.0488 (8)	0.0387 (9)	0.0560 (9)	−0.0052 (6)	−0.0032 (7)	0.0022 (7)
C11	0.0625 (10)	0.0455 (10)	0.0633 (10)	−0.0042 (8)	−0.0060 (9)	−0.0077 (8)
C12	0.0696 (11)	0.0701 (13)	0.0602 (11)	−0.0030 (9)	0.0008 (9)	−0.0162 (9)

### Geometric parameters (Å, °)

**Table:**

O1—C7	1.2215 (18)	C5—C6	1.393 (2)
N1—C7	1.3683 (19)	C5—H5	0.9300
N1—C6	1.3889 (18)	C7—C8	1.476 (2)
N1—C10	1.4629 (19)	C8—C9	1.482 (2)
N2—C8	1.2887 (18)	C9—H9A	0.9600
N2—C1	1.3881 (19)	C9—H9B	0.9600
C1—C2	1.391 (2)	C9—H9C	0.9600
C1—C6	1.397 (2)	C10—C11	1.481 (2)
C2—C3	1.367 (2)	C10—H10A	0.9700
C2—H2	0.9300	C10—H10B	0.9700
C3—C4	1.386 (3)	C11—C12	1.285 (3)
C3—H3	0.9300	C11—H11	0.9300
C4—C5	1.368 (2)	C12—H12A	0.9300
C4—H4	0.9300	C12—H12B	0.9300
			
C7—N1—C6	121.48 (13)	O1—C7—C8	121.81 (14)
C7—N1—C10	117.26 (12)	N1—C7—C8	116.08 (13)
C6—N1—C10	121.20 (12)	N2—C8—C7	123.57 (13)
C8—N2—C1	118.41 (13)	N2—C8—C9	120.44 (14)
N2—C1—C2	118.39 (14)	C7—C8—C9	115.99 (13)
N2—C1—C6	122.20 (13)	C8—C9—H9A	109.5
C2—C1—C6	119.41 (14)	C8—C9—H9B	109.5
C3—C2—C1	120.95 (16)	H9A—C9—H9B	109.5
C3—C2—H2	119.5	C8—C9—H9C	109.5
C1—C2—H2	119.5	H9A—C9—H9C	109.5
C2—C3—C4	119.49 (15)	H9B—C9—H9C	109.5
C2—C3—H3	120.3	N1—C10—C11	114.87 (13)
C4—C3—H3	120.3	N1—C10—H10A	108.6
C5—C4—C3	120.70 (16)	C11—C10—H10A	108.6
C5—C4—H4	119.7	N1—C10—H10B	108.6
C3—C4—H4	119.7	C11—C10—H10B	108.6
C4—C5—C6	120.41 (16)	H10A—C10—H10B	107.5
C4—C5—H5	119.8	C12—C11—C10	127.48 (17)
C6—C5—H5	119.8	C12—C11—H11	116.3
N1—C6—C5	122.71 (14)	C10—C11—H11	116.3
N1—C6—C1	118.25 (13)	C11—C12—H12A	120.0
C5—C6—C1	119.04 (14)	C11—C12—H12B	120.0
O1—C7—N1	122.11 (14)	H12A—C12—H12B	120.0
			
C12—C11—C10—N1	−6.7 (3)		

### Table 1 Offset π–π stacking between the quinoxaline rings.

Cg1 is the centroid of ring N1,C6,C1,N2,C8,C7 and Cg2 the centroid of ring C1–C6.

**Table:**

	Centroid-to-centroid(Å)	plane-to-plane(Å)	offset(°)
Cg1–Cg2^i^	3.8832 (9)	3.509	25.4

Symmetry code: (i) -1+x, y, z.
